# Emerging Microorganisms and Infectious Diseases: One Health Approach for Health Shared Vision

**DOI:** 10.3390/genes15070908

**Published:** 2024-07-11

**Authors:** Maria Vittoria Ristori, Valerio Guarrasi, Paolo Soda, Nicola Petrosillo, Fiorella Gurrieri, Umile Giuseppe Longo, Massimo Ciccozzi, Elisabetta Riva, Silvia Angeletti

**Affiliations:** 1 Operative Research Unit of Laboratory, Fondazione Policlinico Universitario Campus Bio-Medico, Via Alvaro del Portillo, 200, 00128 Rome, Italy; m.ristori@policlinicocampus.it (M.V.R.); m.ciccozzi@unicampus.it (M.C.); e.riva@policlinicocampus.it (E.R.); 2 Unit of Computer Systems and Bioinformatics, Department of Engineering, University Campus Bio-Medico of Rome, Via Alvaro del Portillo, 21, 00128 Rome, Italy; valerio.guarrasi@unicampus.it (V.G.); paolo.soda@umu.se (P.S.); 3 Department of Diagnostic and Intervention, Radiation Physics, Biomedical Engineering, Umeå University, 901 87 Umeå, Sweden; 4 Infection Prevention Control/Infectious Disease Service, Fondazione Policlinico Universitario Campus Bio-Medico, 00128 Rome, Italy; n.petrosillo@policlinicocampus.it; 5 Operative Research Unit of Medical Genetics, Fondazione Policlinico Universitario Campus Bio-Medico, 00128 Rome, Italy; f.gurrieri@unicampus.it; 6 Research Unit of Medical Genetics, Department of Medicine and Surgery, Università Campus Bio-Medico di Roma, Via Alvaro del Portillo, 21, 00128 Rome, Italy; 7 Research Unit of Orthopaedic and Trauma Surgery, Fondazione Policlinico Universitario Campus Bio-Medico, Via Alvaro del Portillo, 200, 00128 Rome, Italy; g.longo@unicampus.it; 8 Research Unit of Orthopaedic and Trauma Surgery, Department of Medicine and Surgery, Università Campus Bio-Medico di Roma, Via Alvaro del Portillo, 21, 00128 Rome, Italy; 9 Unit of Medical Statistics and Molecular Epidemiology, University Campus Bio-Medico of Rome, Via Alvaro del Portillo, 21, 00128 Rome, Italy; 10 Unit of Virology, University Campus Bio-Medico of Rome, Via Alvaro del Portillo, 21, 00128 Rome, Italy; 11 Research Unit of Clinical Laboratory Science, Department of Medicine and Surgery, Università Campus Bio-Medico di Roma, Via Alvaro del Portillo, 21, 00128 Rome, Italy

**Keywords:** emerging infectious disease (EID), emerging microorganisms, newly identified pathogens, one health, artificial intelligence

## Abstract

Emerging infectious diseases (EIDs) are newly emerging and reemerging infectious diseases. The National Institute of Allergy and Infectious Diseases identifies the following as emerging infectious diseases: SARS, MERS, COVID-19, influenza, fungal diseases, plague, schistosomiasis, smallpox, tick-borne diseases, and West Nile fever. The factors that should be taken into consideration are the genetic adaptation of microbial agents and the characteristics of the human host or environment. The new approach to identifying new possible pathogens will have to go through the One Health approach and omics integration data, which are capable of identifying high-priority microorganisms in a short period of time. New bioinformatics technologies enable global integration and sharing of surveillance data for rapid public health decision-making to detect and prevent epidemics and pandemics, ensuring timely response and effective prevention measures. Machine learning tools are being more frequently utilized in the realm of infectious diseases to predict sepsis in patients, diagnose infectious diseases early, and forecast the effectiveness of treatment or the appropriate choice of antibiotic regimen based on clinical data. We will discuss emerging microorganisms, omics techniques applied to infectious diseases, new computational solutions to evaluate biomarkers, and innovative tools that are useful for integrating omics data and electronic medical records data for the clinical management of emerging infectious diseases.

## 1. Introduction

**Figure 1:**
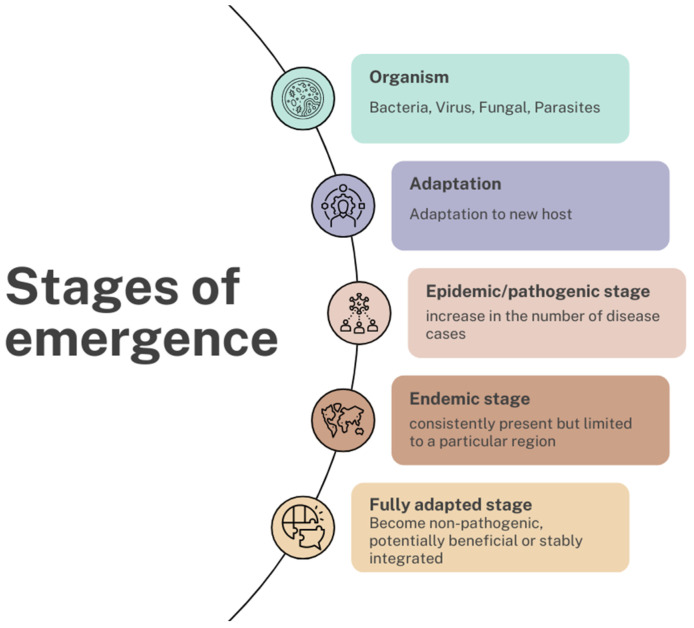
Stages of emergence. The stages of emergency include a phase of adaptation of the organism (such as bacteria, viruses, fungi, and parasites) to the new host, followed by an epidemic/pathogenic phase, where there is an increase in the number of cases; then, there is the endemic phase, where the infectious disease is still present but limited to a particular region, and, finally, the adaptation phase, where the organism can become non-pathogenic and potentially beneficial (e.g., the human microbiota) or be stably integrated into the host genome (e.g., endogenous retroviruses) (created with Canva.com).

The COVID-19 pandemic has emphasized the importance of being able to quickly diagnose new infectious diseases to try to contain the spread of pathogens. With a growing cohort of immunocompromised patients at risk of microbiological infections, attention is being drawn to new possible pathogenic microorganisms. In 2020, the WHO (World Health Organization) reported that the second and third causes of death are also infections [1]. Emerging infectious diseases (EIDs) can be divided into two categories: newly emerging and reemerging infectious diseases. A newly emerging disease could be defined as a new disease that quickly increases in geographic or range incidence or the persistence of infectious diseases that cannot be controlled [2]. Reemerging infections could be due to changes or the evolution of existing microorganisms, historically occurring infections that continue to appear in new locations or in drug-resistant forms, unrecognized infections, or known infections spread to new populations or areas [3]. The National Institute of Allergy and Infectious Diseases identifies the following as emerging infectious diseases: SARS, MERS, COVID-19, influenza, fungal diseases, plague, schistosomiasis, smallpox, tick-borne diseases, and West Nile fever [4]. Reemerging diseases could include malaria, tuberculosis, cholera, pertussis, influenza, pneumococcal disease, and gonorrhea [4]. On the basis of EIDs, there is a continuum of evolution in the interaction between the host, pathogen, and environment. So, the factors that should be taken into account are the genetic adaptation of microbial agents and the characteristics of the human host (e.g., human susceptibility, travel, exposure, and inappropriate use of antibiotics) or environment (e.g., poverty, animal populations, and changing ecosystems). Furthermore, emerging and re-emerging infectious diseases are increasing globally and have an important impact on humans in terms of epidemics and pandemics that threaten to health and global stability. Morens et al. identified successive stages of emergence: (1) organism adaptation to the new host; (2) the epidemic/pathogenic stage; (3) the endemic stage; and (4) the adapted stage (Figure 1) [5]. The adaptive stage could consist of a non-pathogenic stage, in which the organism could also become potentially beneficial to the new host (e.g., human microbiota) or integrate into the host genome (e.g., endogenous retroviruses) [5].

**Figure 2:**
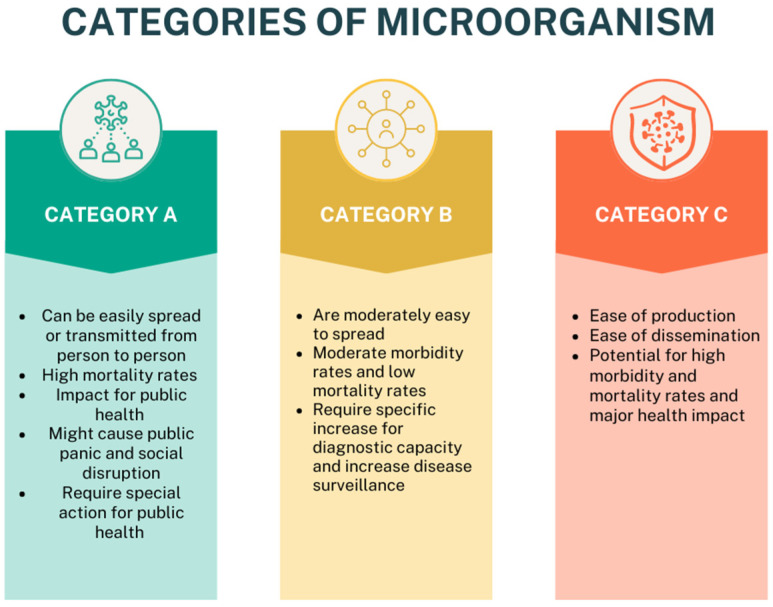
Categories of microorganisms. Category A: agents that pose the highest risk to public and security health, can be easily transmitted from person to person, have high mortality rates, and have an impact on public health; Category B: agents with moderate morbidity and low mortality rates, are moderately easy to spread, require a specific increase in diagnostic capacity, and increase disease surveillance; Category C: agents with high morbidity and mortality rates and ease of dissemination, ease of production, and potential for high morbidity and mortality rates and major health impacts (created with Canva.com).

Microorganisms could be classified into three categories [6]: (1) Category A, agents that pose the highest risk to public and security health; (2) Category B, agents with moderate morbidity and low mortality rates; and (3) Category C, agents with high morbidity and mortality rates and ease of dissemination (Figure 2) [6].

**Figure 3:**
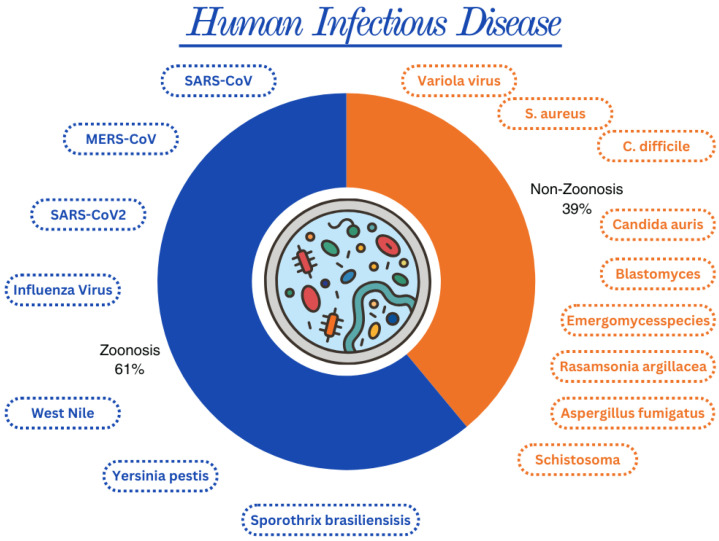
Distribution of transmission of infectious diseases in humans. In human infectious diseases, transmission through zoonosis has a higher impact (61%) and includes coronaviruses, influenza viruses, West Nile virus, *Yersinia pestis*, and *Sporothrix brasiliensis*. On the other hand, non-zoonotic transmissions have a lower impact rate of 39%, including Variola virus, *Staphylococcus aureus*, *Clostridium difficile*, *Candida auris*, *Blastomyces*, *Emergomyces species*, *Rasamsonia argillacea*, *Trichophyton indotinae*, *Aspergillus fumigatus*, and *Schistosoma species* (created with Canva.com).

The types of transmission of infectious diseases in humans could be zoonotic or non-zoonotic. Zoonosis is any infection transmitted directly by animals to humans, and vice versa, through direct contact or through water, food, or other environments [3]. Zoonosis contributes the most to infectious diseases affecting humans (Figure 3) [7]. 

Interspecies transmission plays a key role in the emergence of human infectious diseases. There is a reservoir of “microbial material” that is still unknown and should be discovered and studied. Furthermore, microorganisms have a high adaptive capacity due to their ability to mutate. So, the new approach to identifying new possible pathogens will have to go through genomics-based tests, which are capable of identifying high-priority microorganisms in a short period of time. Furthermore, genomic approaches could be used to create rapid and simple diagnostics, which can help the clinician obtain a fast and accurate infection diagnosis. Moreover, new bioinformatics technologies are enabling data collected during surveillance to be obtained, integrated, and shared globally so that data can be rapidly shared in times of need to guide public health decisions and actions to detect and prevent epidemics and pandemics. We are in a new era of infectious disease surveillance and response. We need to deploy a pandemic early warning and response system that detects microbiological threats in real time to stop infectious diseases before they spread.

## 2. Materials and Methods

### 2.1. Search Strategy

We conducted a review of the literature to evaluate the role of emerging microorganisms and infectious diseases. A search was carried out using the electronic bibliographic databases PubMed and Google Scholar. The research started with studies conducted during and after the COVID-19 pandemic, meaning therefore from 2020 to 2023, using the following terms: “Emerging infectious disease (EID)” or “Emerging microorganisms” or “Newly identified pathogens” or “Emerging Bacteria” or “Emerging Virus” or “Emerging Fungal” or “Emerging Parasites”. Subsequently, we expanded to previous studies before 2020 using the following terms: “One Health” or “Antimicrobial resistance” or “Artificial Intelligence” or “Approach to infectious disease”. All of these articles provided enough information about the relationship between the One Health approach and infectious diseases.

### 2.2. Selection Criteria

The inclusion criteria for study selection comprised the following: (1) observational prospective and retrospective studies, case–control studies, cohort studies, and systematic reviews; (2) studies involving the One Health approach and infectious disease research; and (3) English-written studies. All studies that did not meet the criteria were excluded from the review process.

## 3. Results

### 3.1. Search Strategy

**Figure 4:**
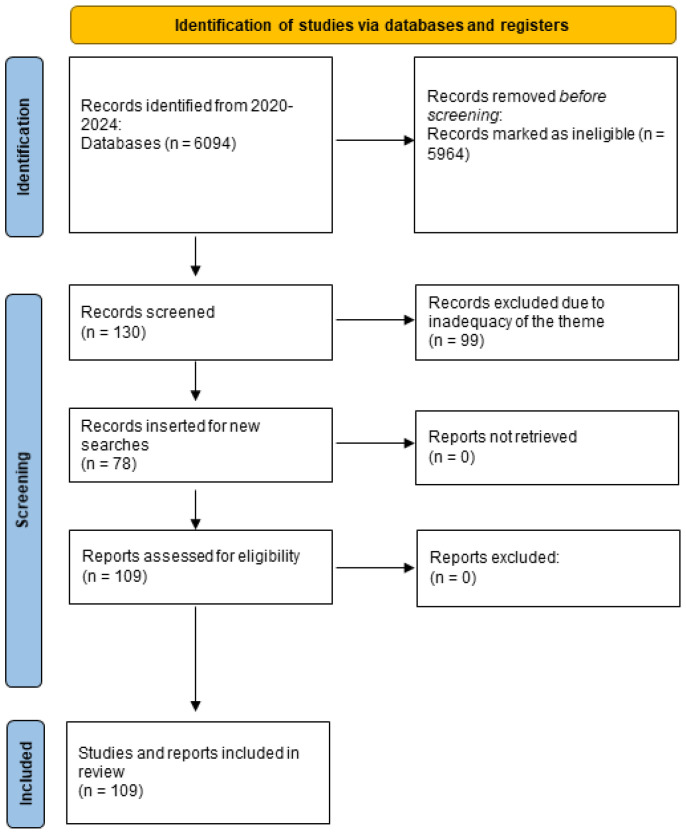
PRISMA flowchart depicting the study design and selection process.

The initial screening produced 6094 studies (Figure 4). One hundred and thirty studies were analyzed, and a total of thirty-one studies initially met the inclusion criteria, including case–control clinical studies, clinical trials, randomized trials, meta-analyses, and systematic reviews. Seventy-eight more articles were subsequently included. The filtering process is depicted in Figure 4, which provides a summary of the included studies.

### 3.2. Emerging Infectious Diseases

Newly emerging infectious diseases are new diseases in which transmission has rapidly expanded to previously naïve populations. The majority of EIDs have a zoonotic origin. Zoonosis manages to jump the species barrier in places and environments where there is greater interaction between humans and animals, increased by ecological changes due to urbanization and climate change [8]. The transmission route could be: (1) direct contact; (2) indirect contact; or (3) vector borne. The National Institute of Allergy and Infectious Diseases identified the following as emerging infectious agents: SARS-CoV, MERS-CoV, SARS-CoV-2, influenza virus, fungal, *Y. pestis*, *S. species*, variola virus, West Nile virus, methicillin-resistant *S. aureus*, and *C. difficile* (Table 1) [4].

### 3.3. Bacterial Infectious Diseases

Bacterial infections have a large impact on public health and are common clinical diseases that can affect different organs and tissues. The transmission in humans is through air, water, food, or living vectors. The principal modes of transmission are contact, airborne, droplet, vectors, and vehicular. Our bodies have different immune mechanisms to respond to infection, such as autophagy, the innate immune response, and the adaptive immune response [36]. They play an important role in the defense against bacterial infections, and the adaptive response, which is directed towards antigens, requires a longer response time. Bacteria have different mechanisms to elude defense responses through autophagy virulence proteins or related molecules [37]. Most of these emerging infections could be due to bacteria that are present in the environment with which humans come into contact [38]. Furthermore, it could be possible that new bacterial infections are due to the inability to quickly identify the new pathogens [39]. One of the most important emerging problems is caused by hospital- and community-acquired antibiotic resistance, such as methicillin-resistant *S. aureus* (MRSA), and bacterial infections, such as *C. difficile*. In fact, the overuse of antibiotics in humans as well as in the food chain, with the changes in the population, contributes to the development of drug-resistant microbes [21]. Some pathogens are re-emerging as multi-drug resistant due to drug resistance mutations, such as drug-resistant malaria [40], drug-resistant tuberculosis [41], and vancomycin-resistant enterococci [42]. It has been determined by increased or inappropriate use of antibiotics. Now, some hospital-acquired resistant organisms are starting to be transmitted at the community level [5]. In the last few decades, a new resistant organism has emerged: plasmid-spread NDM-1 (New Delhi b-lactamase) Gram-negative pan-resistant organisms [43], that produce an enzyme that can destroy many types of antibiotics, including carbapenems, a class of antibiotics used for serious infections. It is due to global antibiotic use and inadequate antibiotic stewardship [43]. Among the re-emerging pathogens, we find *Y. pestis*, which is an obligate parasite and Gram-negative bacterium that caused the plague and killed approximately 200 million people in three major pandemics over the past few centuries [19]. Although the plague may be considered a disease of the past and is now rare, it is still present in many areas, including Africa, Asia, and America, and remains a threat to public health [20]. Some strains of *Y. pestis* exhibit varying degrees of resistance to antibiotics, and, in the last few decades, seven *Y. pestis* isolates with four antibiotic resistance mechanisms have been reported [18]. To reduce the chances of death, it is essential to treat with antibiotics within the first 24 h of the onset of symptoms with streptomycin, gentamicin, tetracyclines, or chloramphenicol [44]. If an antibiotic resistance mechanism is triggered, this can become lethal, as there is no vaccine. Many microorganisms considered pathogens of the past still have notable destructive potential today. The addition of antibiotic resistance coupled with rapid international air travel makes them even more of a real public health danger well into the 21st century.

#### 3.3.1. Antimicrobial Resistance

Bacteria have an astonishing genetic capacity, which allows them to have the plasticity to respond to a wide range of environmental threats, including the presence of antibiotic molecules that could jeopardize their existence.

Antibiotic resistance may be due to antibiotic resistance genes or antibiotic target mutations, which can be intrinsic or diffused through vertical or horizontal inheritance, extrachromosomal plasmids, or mobile genetic elements. Antimicrobial resistance is predominately present in pathogens isolated from people with a clinically significant infection, but it can reside in organisms isolated from asymptomatic people and can trigger the infection when specific conditions arise as in intestinal inflammation [45]. Furthermore, the gut could be a reservoir of antimicrobial resistance, as the use of antibiotics can change the composition of the ecosystem, increasing the opportunity for pathogen growth, a state of “dysbiosis”, which may allow the colonization of antimicrobial resistance due to the increase in antibiotic resistance genes.

#### 3.3.2. Antimicrobial Resistance and the Microbiota Interaction

New studies are focused on the interaction between the microbiota and antimicrobial resistance. Ranallo et al. reviewed the scientific literature exploring the host microbiota and the effects it has on the pathogenesis of infectious diseases and antibiotic resistance [46]. In this context, the USA has made a plan for combating antibiotic-resistant bacteria, which includes microbiome-based products as a possible strategy to reduce the impact of antibiotic resistance [47]. This interaction has just been described in *C. difficile* infection (CDI), one of the most prevalent nosocomial bacteria. In fact, *C. difficile* proliferation could be interfered with by direct or indirect interaction with the microbiota through inhibition of the proliferation of the pathogen [22].

Antimicrobials cause the destruction of the intestinal microbiome, which leads to a reduction in bacterial diversity at the intestinal level, allowing the entry and invasion of pathogens. When diversity is compromised, it can be difficult to increase it [23]. This change in composition and diversity can lead to an entry or increase in resistant microorganisms due to the presence of antibiotic resistance genes [48]. For example, in fecal samples of healthy people, *Prevotella copri* and *Faecalibacterium prausnitzii* were found to be resistant to the cephalosporins ceftriaxone and cefotaxime, with the antibiotic resistance genes near mobile elements such as plasmids [49]. Most Gram-negative gut commensals are intrinsically resistant to quinolones, e.g., *Escherichia coli*, which could acquire quinolone resistance after antimicrobial exposure [50]. Also, *Citrobacter* commensals present in the gut exhibit quinolone resistance, presenting an endogenous reservoir [51].

#### 3.3.3. Antimicrobial Resistance and Bacteriophages

Phage therapy, the use of bacteriophages to treat bacterial infections, gained early interest in the first 20 years of the 1900s but declined with the discovery of penicillin in the 1930s [52]. Modern medicine has revisited phage therapy due to the rise in infections caused by multidrug-resistant bacteria [53]. Phage resistance, similar to antibiotic resistance, poses a challenge to the success of phage therapy. Bacteria have developed various defense mechanisms against phages, some specific to certain species or strains, while others are more common [54]. Phages, unlike antibiotics, have the ability to adapt and deploy their own defense mechanisms to overcome bacterial defenses [55,56,57]. Bacteriophages are highly targeted towards their host, reducing the risk of secondary infections, unlike antibiotics that can influence both pathogens and normal flora, leading to potential superinfections [58]. Additionally, bacteriophages replicate at the site of infection, effectively lysing pathogens, while antibiotics travel throughout the body without concentrating on the infection site [58]. Bacteriophages are environmentally friendly and rapidly identify bacteria, contrasting with the lengthy and costly development process of new antibiotics [59]. Although bacteria can develop resistance to bacteriophages, phage resistance is less concerning than drug resistance and can be countered through phage mutation [60]. Using phage cocktails or combining phage therapy with antibiotics can be synergistic and delay the development of phage resistance [58]. The complexity of phage–bacteria interactions must be understood for successful phage therapy. The impact of defense systems on resistance formation in clinical settings is not fully known, which could hinder the development of effective phage-based therapies.

### 3.4. Viruses

After the COVID-19 pandemic, viruses have received the most attention. In fact, the emergence of viral diseases appeared when an established animal virus switched hosts into humans and was subsequently transmitted within human populations. A virus can acquire the ability to infect new host cells, following which it can adapt to the new host in such a way as to facilitate transmission between hosts through genetic changes that occur in the virus. When the virus acquires the ability to spread epidemically, through changes in the vector or host populations (for example, ecological or environmental changes or increased contact rates), emergence occurs [61].

In the emergence of a viral disease, we can have a single initial infection of a new host without subsequent transmission, a species jump (spillover) that continues to cause transmission in the new host population before the fading epidemic, or an epidemic or sustained transmission of host-to-host endemic diseases in the new host population [62].

The ability of a viral emergency to spread may be due to a series of variables at the viral level, such as viral factors that allow efficient infections in the new host and contact between the reservoir host and the new host. At the host level, there may be barriers at the cellular or organismal level against the infection or environmental determinants that lead to an efficient spread of the virus within the new host population [62].

RNA virus genomes are small, approximately 10–30 kb, with viral mutation rates at the order of 10^−3^–10^−6^ per nucleotide due to no nucleic acid proofreading mechanisms, with the possibility of generating genetically different offspring each time. This error-prone replication can produce viral quasispecies (i.e., large populations of closely related genotypes) [63]. Infection of new hosts may be associated with changes to mutations at the host entry level, i.e., at the binding site of the virus to a specific molecule on the surface of the host cell, achieved through a few nucleotide substitutions [64,65]. For each virus, there is an optimal mutation rate, outside of which lethal mutagenesis or viral clearance can occur. Recombination is the exchange of stretches of genetic material between viruses that are different (but related) and found in the same cell. Generally, for DNA viruses, this phenomenon occurs due to breakage and re-welding of newly synthesized segments, while for RNA viruses, recombination occurs during replication by the jumping of RNA polymerase from one filament to another [66]. A reassortment is a form of recombination that occurs only in viruses with segmented genomes (e.g., orthomyxoviruses, reoviruses, and rotaviruses). In the cell infected simultaneously by two viruses, several segments of both parental viruses with segmented genomes are present. During assembly and maturation, these segments can be randomly distributed in the viral offspring. The result will be virions that possess characteristics that are intermediate between the two parental viruses [67]. These evolutionary mechanisms allow host jumps and immune evasion. In fact, recombinant viruses can differ significantly from parental viruses from an antigenic point of view, hindering the host’s ability to respond immunologically adequately and, from a biological point of view, the ability to invade new replicative niches in different hosts. Reassortment is the basis of the shift in antigen variability that occurs in influenza viruses and the cause of the emergence of pandemic influenza.

In recent years, the incidence of some viruses in human populations appears to be increasing, for example, coronaviruses, influenza viruses, and smallpox. Emerging viruses often have RNA genomes [68], which could be a consequence of the high evolutionary capacity of RNA viruses [69].

Seasonal influenza is one of these emerging viruses, causing highly contagious respiratory diseases and annual epidemics worldwide. Its symptoms could comprise a cold, coughing, fever, chills, muscle aches, and fatigue. The affected organs are the throat, nose, and lungs [13]. One of its complications is pneumonia. The influenza virus is especially dangerous for young or old people or those who have other conditions such as heart disease or asthma [13]. Seasonal influenza may be caused by a new variant that displays minor differences in antigenicity (antigenic drift) from previous strains, meaning that antibodies produced by the previous infection cannot provide strong protective immunity against seasonal variants [14]. The world may also face the threat of an influenza pandemic, caused by the emergence of a new strain of influenza virus with major antigenic changes (antigenic shift) to which humans have never been exposed. There is no pre-existing immunity to them, and they can cause worldwide (pandemic) disease outbreaks. The most recent influenza pandemic occurred in 2009. The new virus was derived from a triple-reassortant North American swine influenza A virus that acquired two viral genes from a Eurasian swine influenza A virus [70].

West Nile virus (WNV) is a type of arbovirus that is spread by mosquitoes belonging to the *Culex* genus. Symptoms of WNV infection can vary from showing no signs at all to experiencing severe neuroinvasive disease that can result in paralysis and death [16]. Although the rise in temperatures in temperate regions will result in more Culex mosquitoes and higher transmission rates of the West Nile Virus (WNV), accurately predicting the risk of infection and its complications for travelers is challenging due to the complex interactions between vectors, pathogens, hosts, and the environment [16,17].

The smallpox virus, an orthopoxvirus, causes the development of a highly contagious disease called smallpox [15,71]. Even though the natural infection has been eradicated, it is among the emerging diseases due to the risk of epidemics linked to bioterrorism [72]. The mortality rate is around 30%, and the disease manifests with severe systemic symptoms and a characteristic pustular rash. Treatment is generally supportive and potentially includes antiviral drugs. Prevention involves vaccination that, due to the associated risks, is limited to selected patients.

In this context, genetic targeting approaches are very promising for identifying new host genes that regulate viral infections, decoding viral life cycles or infectious states, or trying to clarify the functions of viral coded elements by studying genome changes using a genomic approach [73].

John J. Dennehy proposed some possibilities to combat new viral threats, including the development of broad-spectrum antiviral drugs, finding better means to train the immune system to broadly recognize and defend against pathogenic viruses, and computational and bioinformatic tools to identify highly conserved antigenic genes or proteins and their binding epitopes [63].

#### Coronaviruses

Among the coronaviruses (CoVs) that infect humans (HCoV), there are three that could cause mild to moderate upper-respiratory tract illnesses: SARS coronavirus (SARS-CoV), which causes severe acute respiratory syndrome (SARS) and emerged in November 2002; MERS coronavirus (MERS-CoV), which causes Middle East respiratory syndrome (MERS) and emerged in 2012; and SARS-CoV-2, which causes coronavirus disease 2019 (COVID-19) and emerged in 2019 [9].

The SARS-CoV-2 coronavirus pandemic was also due to the high transmissibility and pathogenesis of the virus, in addition to the fact that some infected subjects were asymptomatic or had mild symptoms.

Severe forms of coronavirus disease 2019 (COVID-19) have acute respiratory distress syndrome (ARDS), disseminated coagulopathy, and cardiovascular disorders among their symptoms, and in these cases, the mortality rate is very high [11].

Furthermore, it has been demonstrated that host metabolism plays an essential role in various physiological processes during viral infection; for example, viruses were reported to rewire host cellular lipid metabolism to create replication compartments or lipid accumulation in the lungs of COVID-19 patients and cells infected by SARS-CoV-2 [12].

The state of emergency linked to these infections is due to new variants, and some of these infections, such as MERS-CoV, are still present in some regions of the world, such as the Middle East. Also, the same person could present a coinfection with MERS-CoV and SARS-CoV-2, worsening the patient’s prognosis [10]. Additionally, other CoVs that infect animals can cross species and cause disease in humans [74].

### 3.5. Fungal Diseases

Fungi encompass a broad range of organisms (molds, mushrooms, and yeasts) that can be found in common places such as the soil, air, water, surfaces, and inside our bodies or on our skin.

Mold can worsen breathing problems in people with allergies or asthma, while various types of fungi can infect nails and cause skin rashes. In healthy individuals, fungal infections rarely cause severe health problems, while they can be particularly important in patients with immune deficiencies, such as organ and stem cell transplant recipients, hospitalized patients, or patients with HIV. The therapy employed in these cases involves the use of antifungals, and like with bacteria, fungi are also developing resistance to these classes of drugs. Furthermore, there are no vaccines to prevent fungal infections. We must pay more attention to possible fungal co-infections with other microorganisms, such as respiratory pathogens (influenza or SARS-CoV-2), which can lead to worse outcomes in more fragile patients [25]. New recognitions of fungi are emerging thanks to the advancements in molecular diagnostics, which are enabling the creation of new databases of nucleotide sequences and thus a more accurate taxonomic classification [75]. These new phylogenetic classifications have allowed us to recognize certain fungi as distinct species, such as *R. argillacea*, which was previously classified as *Geosmithia* [76]. We must take into account the fact that many of these new fungi are now considered emerging pathogenic agents, thanks to new phylogenetic classifications [25]. “Consideration of factors contributing to the emergence of fungal pathogens is essential, including the use of new pharmacological immunotherapies that have elevated the risk of opportunistic infections for a greater number of patients”. [77,78]. An example is fungal infections in patients with lung cancer or melanoma undergoing immunosuppressive therapy due to excessive activation of the immune system after treatment with checkpoint inhibitors [79,80]. Among the factors that can cause an emergency, we must also consider the increasing resistance to antifungal drugs compared to the availability of new therapies. Among the mainfungal infections are *Candida* spp., with *C. albicans* being the most common species associated with invasive infections and higher rates of resistance to antifungals [24]. *C. auris* is a species that is attracting attention for its infectious and endemic capabilities, as seen in countries like India and South Africa [81]. Analysis of the whole genome has shown a high number of SNPs compared to the geographic region where they were isolated, as well as a high resistance rate, increasing the urgency [82]. *Blastomyces* is a fungus that can cause infections in humans, blastomycosis, and is more prevalent in some areas of the world, such as America, Canada, and some species in Africa and the Middle East [25]. *Emergomyces* was reclassified as part of the fungi, as it used to be part of the *Emmonsia*-like species [25]. The population at risk for emergomycosis is always immunocompromised or HIV patients [26]. A species to keep under control is *Rasamsonia*; it is very important to initially identify it to implement correct antifungal therapy, as members of the *R. argillacea* species are resistant to isavuconazole and voriconazole [27]. In some regions of Brazil and Argentina, cases of *S. brasiliensis* have been reported due to zoonotic transmission from infected cats to humans [28]. Therefore, even for fungal infections, we must consider the possibility of zoonotic transmissions. Among the emerging fungi, we also consider *Trichophyton*, as there have been outbreaks of dermatophytosis in Asia affecting the trunk and groin, with treatment failures and relapses [29,30]. Among the infectious agents that can cause very invasive and chronic infections is the species *Aspergillus*. In this case, the most exposed group of patients are immunocompromised individuals, patients who have undergone organ transplantation, especially lung transplantation, or patients with lung diseases, as the lungs are the entry route for the microorganism. For this fungus as well, the increase in resistance to treatment is relevant, particularly to azoles, which, in some cases, are the only treatment for these infections. The association of co-infections between *Aspergillus* in patients with respiratory virus infections remains very important, specifically pulmonary aspergillosis associated with influenza (IAPA). In the literature, cases of *Aspergillus* co-infection have been reported in patients with COVID-19 (referred to as COVID-19-associated pulmonary aspergillosis (or CAPA)) [31,32].

### 3.6. Parasites

Parasitic infections have a tendency to emerge in certain areas of the world, greatly influencing the health of the population living in that area. We must also consider that the emergency that may occur in a particular area could become global due to globalization, which brings about a higher likelihood of transmission to people traveling from those areas.

When the host comes into contact with the parasite, infection occurs, and at this moment, the host tries to kill the parasite by activating mechanisms of innate and/or acquired immunity. In the event that the parasite manages to overcome these mechanisms, the parasite survives and establishes a dynamic relationship with the host, becoming an “asymptomatic carrier”. When the balance between the host and parasite is disrupted, the state of illness emerges, and the person becomes symptomatic [83]. It is in the parasite’s interest to try to maintain a balance with the host or evade immune mechanisms in order to complete as many life cycles as possible and, therefore, the possibility of replicating. To achieve this, parasites have developed mechanisms that aim to avoid or manipulate the host’s immune response, such as cellular recognition, activation, and regulation of the host’s innate and adaptive immune responses, causing long-lasting chronic infections [84]. Among the mechanisms to modulate the immune response, we find antigenic variation or prevention of complement activation, inhibition of signaling pathways such as NF-kB, and the release of immunomodulatory molecules that act to suppress the immune response [84]. Furthermore, it has been discovered that parasites use the metabolites or nutrients of the host to their advantage for development and reproduction, releasing molecules that are excretory products of the parasites, which are used to reduce or inhibit the host’s immune response [85]. Among these emerging parasites, we find *S. mansoni*, *S. haematobium*, and *S. japonicum*, which is the etiological agent of intestinal schistosomiasis due to its high mortality, morbidity, and ability to be endemic in sub-Saharan Africa and the Middle East [33]. The life cycle of *Schistosoma* begins when the eggs containing miracidia are excreted in freshwater through feces or urine, where they hatch and release miracidia under optimal conditions. When these miracidia encounter *Biomphalaria* spp., they enter the snail, which becomes the intermediate host. The miracidia undergo successive divisions, and after two generations of sporocysts, they become cercariae, which are released by the snails into the water. When these cercariae encounter a human host, they penetrate through the skin. During penetration, the cercariae lose their bifurcated tail, transforming into schistosomules that migrate through the venous circulation to the lungs, then to the heart, and then develop in the liver, exiting the liver through the portal vein system when they are mature [34]. Mature females deposit eggs, many of which are expelled from the body through feces or urine, while some eggs remain in the body and are deposited in host tissues where they are surrounded by immune cells that form structures called granulomas. These destroy the eggs and neutralize egg antigens that would otherwise be pathogenic, but they also cause fibrinogenesis, which is the main cause of infection pathology [35]. The impact of this parasite on human health is significant, which is why it is important to find strategies to try to contain its spread.

One aspect that we must also consider is the possibility of co-infections with other pathogens, such as SARS-CoV-2. In fact, there have been reported cases of co-infection with parasitic diseases and COVID-19, leading to a resurgence or relapse of disease caused by the parasites. Additionally, some symptoms can be similar between certain parasites and COVID-19, making diagnosis and treatment even more challenging [86]. A systematic review of the literature has examined all studies in which there has been a co-infection between parasites and COVID-19, and controversy has emerged regarding the effect of co-infection on the outcome of COVID-19. Parasite co-infection may inhibit the effective immune response to COVID-19 or may also suppress immune responses and mitigate vaccine effectiveness. The fact that many people infected with SARS-CoV-2 receive immunosuppressive drugs represents a possible risk factor for severe parasitic infections [87]. To try to decrease or limit the spread of these infections, it is always essential to have an accurate and rapid diagnosis [88].

## 4. Approaches to Control Infectious Disease

The Koch criteria provided a rational scientific method for the study of infectious diseases and changed microbiology and medicine. However, the postulates have some limits that he identified immediately. In the last 30 years, new genomic approaches have been developed, and many unknown bacteria and viruses have been detected in sick and healthy individuals. David N. Fredericks and David A. Relman revised Koch’s postulates in the era of genomics [89]: (1) A nucleic acid sequence belonging to a putative pathogen should be present in most cases of infectious disease, and preferentially in those organs or anatomical sites known to be affected by the disease. (2) The copy numbers of pathogen-associated nucleic acid sequences should be lower or absent in non-diseased hosts or tissues. (3) As the disease resolves, the copy numbers of pathogen-associated nucleic acid sequences should decrease or become undetectable. Conversely, in cases of clinical relapse, the opposite should occur. (4) When the nucleic acid sequence is detected before the disease occurs, or the number of copies of the sequence correlates with the severity of the disease or pathology, the association between the disease and the sequence is more likely to be causal. (5) The nature of the microorganism inferred from the available sequence should be consistent with the known biological characteristics of the group of microorganisms to which it belongs. (6) Correlates of the sequence identified in tissues should also be sought in cells: attempts should be made to demonstrate specific in situ hybridization of the microbial sequence in areas where tissues are pathological and where microorganisms are visible or in areas where it is presumed that microorganisms are found. (7) This evidence of causality based on microbial sequences should be reproducible.

Three main approaches need to be kept in mind in emerging disease infection: (1) the development of new diagnostic tools; (2) an increase in human exposure to bacterial pathogens; and (3) the emergence of more virulent bacterial strains.

### 4.1. Multi-Omics Approaches to Infectious Disease for One Health Vision

**Figure 5:**
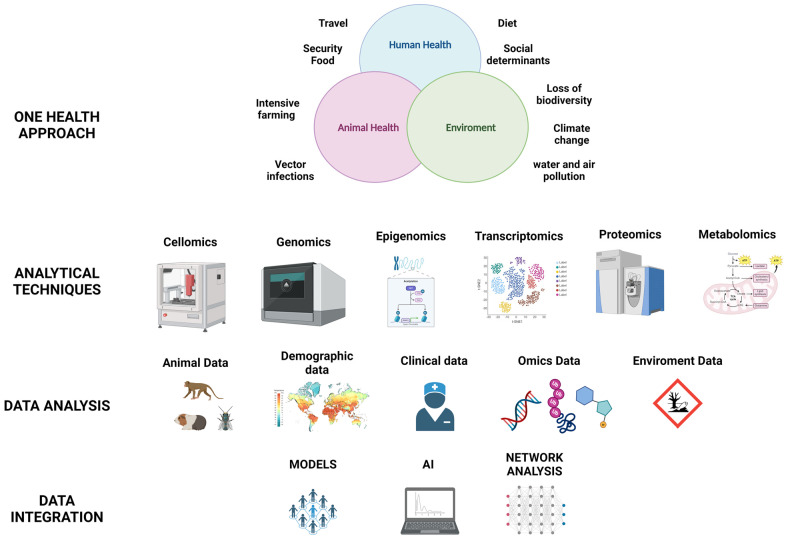
The One Health approach and omics techniques (created with BioRender.com). An integrative approach could be a new strategy for in-depth profiling in infectious diseases, combining data from the One Health approach and analyzing these data with different platforms: genome sequencing (next-generation sequencing—NGS), proteomics, metabolomics through liquid chromatography–mass spectrometry and gas chromatography–mass spectrometry (LC–MS or GC–MS), or even from metabolomic data obtained from nuclear magnetic resonance (NMR) experiments. With this amount of data, there is a need for an analysis of individual data first and then proceeding to an integrated analysis, which can allow the creation of models, network analysis, and the use of AI.

The COVID-19 pandemic has highlighted the urgent need for a One Health approach to control and prevent future pandemics, which includes the development and implementation of early warning systems that are dynamic and based on a One Health approach for emerging infectious diseases [90]. In fact, health threats require prevention and preparedness, posing various challenges such as identifying and evaluating risks as soon as possible, evaluating multifactorial factors, engaging with stakeholders, and making data easily accessible in a crosscutting and transparent manner. We cannot simply reduce any pandemic to a single triggering factor without taking into account the multiple causes, complex determinants, and contributing factors that have led to its occurrence. Up to now, the main approach to epidemics and pandemics has been to only control health risks once they emerge or re-emerge. Instead, a much more effective strategy would be to promptly assess the potential risk factors involved in order to prevent or mitigate their consequences [91]. From this perspective, the One Health approach represents a valid health and scientific strategy. The One Health holistic approach, which is a healthcare model based on the integration of different disciplines, is founded on the recognition that human health, animal health, and ecosystem health are inseparably interconnected [92]. The One Health approach is ideal for achieving global health because it allows us to address the needs of the most vulnerable populations based on the relationship between human health, animal health, and the environment in which they live. This approach allows us to take into consideration the spectrum of determinants that emerge from this relationship. This approach involves integrating data from human health (diet, social determinants, or travel), animal health (intensive farming or vector infections), and the environment (loss of biodiversity, deforestation, climate change, or water and air pollution) [93,94]. Samples from the three areas (human health, animal health, and the environment) can be investigated through the use of omics and meta-omics disciplines, which currently allow for a comprehensive study starting from a wide range of matrices, such as biological fluids, tissues, or environmental data. In fact, omics sciences provide a holistic view of the system under study, surpassing the classical approach of genetics/biochemistry studies based on one or a few target molecules. These approaches allow for obtaining complete profiles of biological “features” (genes/transcripts/proteins/metabolites) to achieve a broad description of a biological system by the so-called systems biology. The omics technological approach and advancements in computational levels allow for a deep multi-omics phenotyping, enabling a more in-depth analysis of the interaction between multiple biological levels, facilitating precision healthcare approaches (Figure 5). Multi-omics integrative approaches are useful in healthcare as they can allow for disease subtyping, predict disease risk, stratify patients for therapy, identify biomarkers, and extract biological information. In the field of infectious diseases, many omics approaches have been implemented for the COVID-19 pandemic. For example, a study aimed to understand the dynamics of circulating immune cells in patients with COVID-19 through a longitudinal multi-omics analysis of peripheral blood mononuclear cells (PBMCs) throughout the course of the disease. The omics data obtained showed the cellular effects caused by the infection both at epigenetic and transcriptional levels, useful for developing biomarkers and precision treatments for COVID-19 [95]. One of the studies utilizing omics platforms and COVID-19 involved integrated single-cell multi-omics profiling of lung tissues from patients, identifying over 1000 genes as risk factors for severe COVID-19 in cells. This study also integrated the analysis with a machine learning algorithm, aiming to predict severe disease in non-elderly patients based on a Genome-Wide Association Study (GWAS) and single-cell omics [96].

### 4.2. Genomic Sequencing for Outbreaks

In the field of the prevention and diagnosis of infections caused by pathogens, the use of next-generation sequencing (NGS) techniques is helpful as they allow for a more in-depth knowledge of the pathogen genome for diagnosis and therapeutic approaches in infectious diseases. NGS techniques can be very useful in characterizing emerging pathogens, evaluating antimicrobial resistance genes (ARGs), assessing virulence factors, or monitoring pathogens that are at higher risk. Among the most commonly used approaches in this field, we find metagenomics, which allows for massive and random sequencing of DNA fragments, enabling genomic fragments to be sampled, sequenced, and annotated almost in real time. The fields of application of NGS techniques are (1) targeted NGS with various enrichment methods, including probe hybridization or amplification (i.e., bacterial profiling of 16S rDNA or PCR amplification of other specified targets followed by NGS); (2) whole genome sequencing (WGS) of a pathogen of interest (used for phylogenetic analysis, identification of virulence or resistance genes, or identification of new species); or (3) unbiased next-generation metagenomic sequencing (mNGS) that allows for the analysis of pathogens that are difficult or unable to be cultured (such as viruses), directly from clinical samples [97]. The mNGS approach can allow for the identification of taxonomic profiles in polymicrobial infections, the identification of DNA and RNA viruses, antimicrobial resistance from complex matrices, the identification of antiviral resistances, and, ultimately, the surveillance of infections and epidemics. Among these NGS approaches, shotgun sequencing is the one that is being sought to be used, as it allows us to obtain information, in the same analysis, of all microorganisms present in the sample (bacteria, viruses, fungi, and parasites). It is possible to decide to start with just DNA or to work with both DNA and RNA, depending on the needs and which microorganisms you want to identify. Computational analysis of the obtained sequences is important for the identification of microorganisms through alignment of the obtained sequences with known sequences in the databases [98].

### 4.3. Epigenomics for Outbreaks

Among the One Health approaches, there is epigenetics, which helps us understand the differences in the acquisition, manifestation, and outcome of infections in the population. Histone modifications, non-coding RNA, and DNA methylation deepen the host–pathogen interaction, as they regulate both pathogen and host genes. For example, the host may silence the viral genome through DNA methylation, or viruses may modify specific host histones to make the host genome more accessible for viral replication [99]. Indeed, DNA methylation leads to epigenetic changes as an immune response to pathogens by regulating proteins such as DNA methyltransferase. When cells are exposed to pathogens, DNA methylation can occur quickly in an attempt to remove the pathogens [100].

### 4.4. Proteomics for Outbreaks

Proteomics also proves to be an optimal strategy in approaching infectious diseases and emerging diseases, as proteins can have various functions, can be utilized in disease identification and pathogenesis, and may potentially become targets for antiviral drugs. The study of proteins has been made possible through mass spectrometry, which has also allowed us to reveal key protein–protein interactions and discover signaling networks in infectious diseases [101]. Sperk et al. conducted a literature review, providing an overview of the strategies used in proteomics in infectious diseases. The most commonly used approach is data-dependent acquisition (DDA), which allows for the scanning of peptides that are fragmented and then analyzed. Other approaches include label-free quantification (LFQ), multiplexing with isobaric labeling, selected reaction monitoring (SRM), and data-independent acquisition (DIA) [101]. These approaches have revolutionized microbiology and virology to try to identify new pathogens, understand signaling pathways, host responses to pathogens, and host–pathogen interactions.

### 4.5. Metabolomics for Outbreaks

Another approach that has been explored and found to be important in understanding host–pathogen interactions is metabolomics, a discipline that studies small molecules. In many studies, metabolomics is now being associated with other omics disciplines, such as genomics, metagenomics, proteomics, or transcriptomics, to try to achieve a more comprehensive view of the system. It is also used in the search for biomarkers that can be used for a diagnosis or prognosis. The approaches used are based on mass spectrometry or nuclear magnetic resonance spectroscopy (NMR). In the literature, the role of metabolomics in the field of infectious diseases is being investigated, as it can also allow for the identification of new markers in the treatment of infections. A literature review sought to delve into the role of metabolomics in infectious diseases. For example, in Dengue, it has been observed that the host cell attempts to balance polyunsaturated phospholipids to regulate inflammation. In COVID-19, markers of inflammation have been identified, such as an increase in the kynurenine/tryptophan ratio, an increase in the α-1-acid glycoprotein signal, and the modulation of lipid profiles (high- and low-density lipoproteins and triglycerides). In bacterial infections, it is seen that lipopolysaccharide (LPS) stimulates macrophages towards an inflammatory response [102].

### 4.6. Data Integration Omics

From each omics platform—genomics, proteomics, metabolomics, and transcriptomics—a vast amount of data is generated. These data require meticulous integration steps to provide a comprehensive picture of the disease phenotype within the context of the system. The integration process combines gene, protein, metabolite, and transcript information using various methodologies that are crucial for understanding the complex interactions that define a disease state. The most commonly used approaches for this purpose include statistical integration, model integration, network integration, and data integration. These methods aim to consolidate omics data into a unified analysis framework, enhancing our ability to interpret complex biological phenomena [103]. As methods for data integration, meta-dimensional analysis and multi-stage analysis are particularly noteworthy. Meta-dimensional analysis involves combining all data through concatenation or transformation into a simultaneous matrix, effectively allowing for comprehensive cross-omics analysis. Multi-stage analysis, on the other hand, utilizes two numerical or categorical characteristics of the data to explore relationships and patterns that may not be apparent from single-omics analysis [104]. Various algorithms, such as network analysis, Bayesian analysis, clustering, and data reduction techniques, are employed to manage and interpret these integrated datasets. These algorithms help identify significant patterns and correlations that can lead to new insights into disease mechanisms [105]. In order to increase the capacity of an algorithm beyond the data obtained from omics platforms, it is also essential to provide clinical and epidemiological data on the disease. This integration enriches the dataset, providing a more accurate and holistic view of the disease context. It is important to take into consideration some key points, such as data generation, acquisition, analysis, and visualization. Each of these stages presents unique challenges and requires careful consideration to ensure data integrity and meaningful interpretation. Addressing these aspects can significantly enhance the robustness of the findings and the utility of the integrated data in disease research [106].

### 4.7. Artificial Intelligence and Clinical Decision Support System in Outbreaks

Artificial intelligence (AI) and machine learning (ML) technologies are revolutionizing the surveillance and management of infectious diseases. These tools provide the ability to quickly analyze vast amounts of data, predict potential outbreaks, and offer actionable insights, significantly advancing global health security efforts.

As discussed in the work of Oeschger et al. [107], AI is pivotal in developing early warning systems that can detect novel pathogens before widespread transmission occurs. This study emphasized the use of AI to analyze various factors, including climate variations and zoonotic spillovers, to pinpoint regions and species at elevated risk of infection. Additionally, AI technologies help monitor health records and public data to spot outbreaks faster than traditional methods, thus facilitating earlier and more effective interventions. Bess et al. [107] highlighted the critical role of ML in accelerating the discovery of novel antimicrobial agents. These AI-driven approaches streamline the identification process of potential drug candidates by analyzing complex biological data and predicting interactions at a scale and speed unachievable by human capabilities alone. This rapid discovery process is essential for addressing the dynamic challenge of microbial resistance. Reel et al. [108] illustrated how ML methods integrate and analyze multi-omics data (genomics, proteomics, and metabolomics) to provide a comprehensive understanding of diseases. This integration facilitates the discovery of new biomarkers, enhancing disease prediction, patient stratification, and the delivery of precision medicine. The insights gained from such analyses are crucial for developing targeted interventions and understanding the complex dynamics of infectious diseases. According to Peiffer-Smadja et al. [109], AI and ML are increasingly being employed in clinical decision support systems to improve decision-making in infectious disease management. These systems analyze complex and voluminous clinical data to provide real-time recommendations, improve diagnosis accuracy, and predict patient outcomes. This review also underscored the importance of expanding AI applications in diverse healthcare settings, including low- and middle-income countries, to enhance the global applicability and effectiveness of these tools.

Despite these promising advancements, the deployment of AI in public health faces several challenges, including ethical concerns, data privacy, and the need for interpretable and trustworthy models. Addressing these issues requires ongoing research, multidisciplinary collaboration, and rigorous validation to ensure that AI tools are both effective and equitable across different populations and regions. Integrating AI into infectious disease management offers a proactive approach to predict, detect, and respond to outbreaks. This shift towards data-driven public health strategies enhances the ability to manage and mitigate the impact of infectious diseases globally. However, the success of such technologies will depend on their continuous improvement and adaptation to meet the evolving challenges of public health.

## 5. Conclusions: Creating a Driven, Shared Vision for Health

In conclusion, we can affirm that the approach to emerging infectious diseases requires expanding translational research through an approach that brings together all the systems involved (human health, animal health, and the environment) to try to develop new approaches for the prevention, diagnosis, and treatment of infectious diseases, addressing all emerging challenges. As we have seen, omics approaches are enabling us to deepen our understanding of the possible etiology and pathogenesis of infectious diseases in order to be prepared for emerging microorganisms and their related diseases. It will certainly be necessary to supplement these data with the analysis of determinants and the distribution of pathogens, which comes from macroecology, allowing us to develop models that seek to predict and respond to environmental changes. By combining all non-omics data (demographic data, electronic medical records, and laboratory data), omics data (genomics, transcriptomics, proteomics, and metabolomics), and environmental data (from macroecology) and feeding them into artificial intelligence, it will be possible to create models that can be quickly queried by specialists for early diagnosis of a new infection. This approach will help in containment and in discovering new therapeutic approaches to prevent harm to public health.

In conclusion, the following fundamental steps are necessary:

- Collect samples and data on human, animal, and environmental health;
- Analyze the samples through omics platforms;
- Integrate all the obtained data;
- Create artificial intelligence models that can be queried;
- Implement protocols and approaches, including instrumental ones, to be prepared for possible new infections;
- Deepen and study all the gaps that still exist in our knowledge about the circulation of microorganisms between humans, animals, and the environment.

## Figures and Tables

**Table 1:** Newly emerging diseases.

Agent	Type of Microorganism	Transmission	Human Disease	Zoonotic or Non-Zoonotic	Reference
**Virus**					
SARS-CoV	Virus	Droplets and aerosols	Severe acute respiratory syndrome	Zoonotic	[9]
MERS-CoV	Virus	Droplets and aerosols	Middle Eastern respiratory syndrome	Zoonotic	[10]
SARS-CoV-2	Virus	Droplets and aerosols	Acute severe coronavirus 2 respiratory syndrome	Zoonotic	[9,11,12]
Influenza viruses	Virus	Droplets and aerosols	Respiratory disease	Zoonotic	[13,14]
Variola virus	Virus	Droplets or direct contact	Smallpox	Non-zoonotic	[15]
West Nile	Virus	Bitten by a infected mosquito	West Nile Fever	Zoonotic	[16,17]
**Bacteria**					
*Y. pestis*	Bacterium	Bitten by a rodent flea	Plague	Zoonotic	[18,19,20]
*S. aureus*	Bacterium	Contact contagion	Skin infections and sometimes pneumonia, endocarditis, and osteomyelitis	Non-zoonotic	[21]
*C. difficile*	Bacterium	Fecal–oral	Pseudomembranous colitis	Non-zoonotic	[22,23]
**Fungi**					
*C. auris*	Fungi	Direct contact	Infections are generally low-level; however, in immunocompromised individuals, it can cause serious infections and presents a high lethality in invasive forms	Non-zoonotic	[6,24]
*Blastomyces*	Fungi	Inhalation	Atypical and disseminated blastomycosis in immunocompromised humans and companion animals. Cases reported in western states and provinces of the US and Canada	Non-zoonotic	[25]
*E. species*	Fungi	Breathing	Disseminated infections in patients with advanced HIV/AIDS. Systemic infections in other immunocompromised patients	Non-zoonotic	[25,26]
*R. argillacea*	Fungi	Unknown	Chronic granulomatous disease, hematologic malignancies, and colonization in cystic fibrosis patients. Intrinsic resistance to voriconazole and isavuconazole	Non-zoonotic	[27]
*S. brasiliensis*	Fungi	Cat-transmitted	Zoonotic transmission can occur with outbreaks in humans reported due to infected cats	Zoonotic	[28]
*T. indotinae*	Fungi	Sexual contact	Outbreaks of dermatophytosis with emerging resistance to terbinafine, fluconazole, and griseofulvin in patients in northern India, leading to clinical failures in the treatment of tinea corporis/cruris infections	Non-zoonotic	[29,30]
*A. fumigatus*	Fungi	Droplet or airborne	Pulmonary aspergillosis, allergic bronchopulmonary aspergillosis, invasive aspergillosis, and superficial aspergillosis	Non-zoonotic	[31,32]
**Parasite**					
*Schistosoma mansoni*	Parasite	Contact-contaminated water	Schistosomiasis	Non-zoonotic	[33,34,35]
*Schistosoma* *haematobium*	Parasite	Contact-contaminated water	Schistosomiasis	Non-zoonotic	[33,34,35]
*Schistosoma* *japonicum*	Parasite	Contact-contaminated water	Schistosomiasis	Non-zoonotic	[33,34,35]

## Data Availability

No new data were created or analyzed in this study. Data sharing is not applicable to this article.
